# Influence of Practice Environment and Manager Leadership Style on Interprofessional Collaboration and Quality of Care in Acute Care Nurse Practitioners

**DOI:** 10.1097/jnr.0000000000000740

**Published:** 2026-05-08

**Authors:** Sheng-Shiung HUANG, May-Pin CHEN, Shiow-Luan TSAY

**Affiliations:** 1 College of Nursing and Health Sciences, Da-Yeh University, Changhua, Taiwan; 2 Department of International Medicine, Ditmanson Medical Foundation Chiayi Christian Hospital, Chiayi, Taiwan

**Keywords:** nurse practitioners, practice environment, leadership styles, interprofessional collaboration, quality of care

## Abstract

**Background:**

Over 90% of nurse practitioners (NPs) are employed in hospitals in Taiwan. The influence of different practice environments and manager leadership styles on NP interprofessional collaboration and quality of care in acute care hospitals has been inadequately studied.

**Purpose:**

This study was designed to investigate the influence of practice environment and leadership style on NP interprofessional collaboration and the quality of care they provide in acute care settings.

**Methods:**

A cross-sectional design and a national online survey were employed to collect data from 1,198 NPs who are members of the Taiwan Association of Nurse Practitioners (TANP). The measures utilized in this study include the Nurse Practitioner Acute Care Organizational Climate Questionnaire, the Multifactor Leadership Questionnaire–Form 6S, and the Provider-Perceptions of Team Effectiveness Questionnaire (Provider-PTE). A multiple regression model was applied to identify the factors potentially associated with interprofessional collaboration and quality of care.

**Results:**

Physician relations and professional visibility were identified as the two most critical factors within the practice environment, enhancing interprofessional collaboration and quality of care, and “management with expectation” was identified as a key leadership strategy for improving both outcomes. These three factors accounted for 44.8% and 30.6% of the respective variances in NP interprofessional collaboration and quality of care.

**Conclusions/Implications for Practice:**

Improving practice-environment factors such as relationship with physicians and professional visibility, as well as managing NPs using an expectations-based leadership style, offer the potential to significantly enhance NP interprofessional collaboration and the quality of care they provide. Health care organizations may consider developing policies that focus on improving the practice environment as well as implementing transactional leadership styles to promote NP interprofessional collaboration and the quality of care they provide.

## Introduction

Acute care hospitals in Taiwan increasingly employ nurse practitioners (NPs) to address the shortage of medical staff and the growing health care demands of the population. Today, 90% of the more than 12,000 NPs employed in Taiwan work in acute care settings (Taiwan Association of Nurse Practitioners [TANP], 2025). NPs significantly contribute to acute care environments, particularly in terms of enhancing clinical patient safety and improving care outcomes (Barnett et al., 2022; Carranza et al., 2020). Furthermore, NPs provide high-quality health care, alleviating health care system burdens (Huang et al., 2024).

Taiwan’s health care system is similar to the restricted practice model, outlined by the American Association of Nurse Practitioners, used in the United States (AANP; 2026). Under this model, NPs practice under physician supervision (Huang et al., 2024), with NPs managing patient care, prescribing medications, and providing treatment in collaboration with physicians. Thus, interprofessional collaboration is essential for NPs to provide clinical care to patients effectively.

Interprofessional practice, teamwork, and collaboration are collectively referred to as interprofessional collaboration, a term that may be broadly defined as the processes or actions employed by team members to work together to achieve mutual goals (Kilpatrick et al., 2019). The interprofessional collaboration process often encompasses decision-making, communication, cohesion, care coordination, problem-solving, and a focus on patients and their families. These elements are fundamentally grounded in role clarity and trust, particularly among physicians and NPs (Kilpatrick et al., 2019, 2021). Interprofessional collaboration among health care team members is essential for the provision of high-quality medical care (Lin et al., 2024) and has been shown to enhance patient outcomes, especially through improved communication and collaboration between physicians and NPs (Alkhaqani, 2022; Kilpatrick et al., 2021). Therefore, a comprehensive exploration of the influence of the practice environment on interprofessional collaboration may provide insights into how to better facilitate collaboration between NPs and team members and thus further improve patient outcomes.

In addition to interprofessional collaboration, quality of care is a critical and common metric used to assess NP contributions to the health care system (Lucatorto & Walsh-Irwin, 2020). NPs play an essential role in patient care that is closely linked to the quality of care and patient outcomes. The findings of Aiken et al. (2021) indicate that teams characterized by a greater representation of NPs within inpatient care settings tend to provide a relatively higher quality of care and achieve better safety outcomes compared with teams with a lower number of NPs. Moreover, Barnett et al. (2022) reported the quality of care delivered by NPs to be comparable to that delivered by physicians. Similarly, Carranza et al. (2020) indicated that NPs can achieve optimal health outcomes and quality of care. However, there remains a significant gap in data related to the quality of care provided by NPs in Taiwan.

Practice environment is often used synonymously with organizational climate, which reflects the organizational attributes of a workplace that either facilitate or hinder individual performance (Pereira et al., 2024). In the context of nursing care, the practice environment includes aspects such as physician relations, professional visibility, administrative relations, and social support for independent practice (Poghosyan et al., 2019). The majority of NPs in Taiwan work in acute care settings (TANP, 2025), and optimizing the practice environment in high-demand settings is known to be essential in improving patient care outcomes (Huang et al., 2024). Previous studies have established a connection between practice environment and interprofessional collaboration (Donelan et al., 2020; Pérez-Vallejo & Fernández-Muñoz, 2020), with Pérez-Vallejo and Fernández-Muñoz (2020) demonstrating a significant correlation (*p*<.01). Moreover, a secondary analysis conducted by Donelan et al. (2020) revealed heightened perceptions of effective teamwork to be significantly associated with a supportive practice environment. Also, Luo et al. (2021) noted that NPs who receive sufficient support from their practice environment are better equipped to deliver effective health care. The current body of evidence indicates that favorable practice environments assist team members in understanding and recognizing the professional role of NPs and help enhance interprofessional collaboration. However, most of the findings to date draw on non-clinical samples and secondary data analysis methods, with limited research examining the impact of the practice environment on NP interprofessional collaboration efficacy in acute care settings.

The results of prior research have also established a correlation between practice environment and quality of care provided (Abraham et al., 2021; Poghosyan, Ghaffari, Liu, McHugh, 2020). For example, Ho et al. (2021) identified the positive effects of supportive practice environments on the professional performance of NPs and their practice outcomes. Similarly, Abraham et al. (2021) suggested positive practice environments may have the potential to improve the effectiveness of NP in clinical settings, consequently leading to an enhancement in the quality of patient care. Furthermore, Poghosyan, Ghaffari, Liu, and McHugh (2020) indicated that greater organizational-level support is associated with improved quality of care. However, despite the substantial research focus on the relationship between various aspects of the practice environment and quality of care, no national survey of NPs has been conducted that specifically examines this relationship.

The leadership style practiced by their direct managers significantly influences NPs in their pursuit of common health care objectives, particularly in the realms of individual motivation and interprofessional collaboration (Hsu et al., 2022). In Taiwan, NPs are required by their health care organization’s guidelines and public policy to engage in collaborative partnerships with physicians. As such, management leadership style is an important factor of influence on interprofessional collaboration efficacy. NP-management strategies that emphasize empowerment and facilitate communication can enhance and integrate the expertise of both NPs and physicians, leading to more effective collaboration (Folkman et al., 2019; Huang et al., 2024).

Effective leadership has been posited as essential to promoting and sustaining interprofessional collaboration (Moilanen et al., 2020), particularly in the context of addressing nursing-care-related challenges such as supportive practice, excessive workload (Ystaas et al., 2023), and rapidly changing needs (Im et al., 2024). Also, supportive leadership may alleviate negative correlations with turnover intentions (Tolksdorf et al., 2022) and improve patient outcomes (Bouton et al., 2023) and clinical performance (Allan & Rayan, 2023).

The influence of leadership has the potential to cultivate interpersonal relationships and encourage trust and interdependence among team members in practice (Lin et al., 2024), both of which are essential to interprofessional collaboration. The association between leadership and interprofessional collaboration has been examined by previous researchers (Hsu et al., 2022; Moilanen et al., 2020; Ystaas et al., 2023). Ystass et al. noted that effective leadership significantly influences the successful implementation of interprofessional collaboration in patient care, suggesting that appropriate leadership may enhance interprofessional collaboration. Folkman et al. (2019) emphasized the impact of leadership on the roles of various health care professions and the dynamics of interprofessional collaboration. Evidence presented in Moilanen et al. (2020) further establishes a strongly positive relationship between leadership and interprofessional collaboration in health care settings. In addition, Hsu et al. (2022) found that leadership, especially the idealized influence style of leadership, positively enhances the performance of team-based practices. However, prior research has predominantly conceptualized leadership as a singular independent variable, focusing particularly on the transformational leadership style and not differentiating among various leadership styles. The effects of leadership style on interprofessional collaboration need to be examined further using a nationally representative sample.

The significant influence of effective leadership on the quality of care has been well documented. Leadership helps enhance clinical competence by promoting critical thinking and facilitating effective decision-making processes (Lin et al., 2024), which, in turn, empower NPs to deliver care that is both accountable and responsible, thus improving the overall quality of patient care (Hu et al., 2025). Research indicates that managers who exhibit effective leadership qualities are capable of inspiring positive behaviors and improving practice performance (Alanazi et al., 2023; Allan & Rayan, 2023; Gebreheat et al., 2023; Ystaas et al., 2023). Furthermore, hospital supervisors are encouraged to adopt appropriate leadership styles tailored to various situations and nursing professionals that can promote better health care quality (Im et al., 2024; Ystaas et al., 2021). The findings of a recent systematic review highlight a significant association, both direct and indirect, between quality of patient care and the transformational leadership style of managers (Alanazi et al., 2023). In addition, Gebreheat et al. (2023) affirmed the transformational leadership style to be vital in enhancing nursing care quality. Conversely, evidence from a recent review suggests that inappropriate leadership styles (e.g., toxic leadership) can impact quality of care negatively (Labrague, 2021). Although the relationship between leadership and quality of care has been previously explored, the focus has been mainly on the influence of the transformational leadership style. However, the effects of different leadership styles (e.g., transactional leadership) on the quality of care provided by NPs remain unclear.

Certain demographic and practice-related variables may also influence interprofessional collaboration and quality of care. For example, age, gender, hospital level, daily patient load, years of practice experience, and annual salary have been identified as potential factors of influence on these two variables (Carthon et al., 2022; Poghosyan, Ghaffari, Liu, & McHugh, 2020).

In summary, the extant research targeting practice environments, leadership styles, interprofessional collaboration, and quality of care inadequately addresses NPs. These prior studies have used non-NP samples (Moilanen et al., 2020; Pérez-Vallejo & Fernández-Muñoz, 2020), small sample sizes (Abraham et al., 2021; Kilpatrick et al., 2021; (Poghosyan, Ghaffari, Liu, & McHugh, 2020), secondary analysis (Abraham et al., 2021; Alanazi et al., 2023; Carthon et al., 2022; Donelan et al., 2020; Ystaas et al., 2023), and review designs (Barnett et al., 2022; Carranza et al., 2020). Furthermore, practice environments, leadership styles, interprofessional collaboration, and quality of care, specifically among NPs in acute care settings, have yet to be concurrently investigated in the literature. In response to the abovementioned gaps in the literature, the objective of this study was to explore, using a national survey, the influence of different practice environments and leadership styles on NP interprofessional collaboration efficacy and the quality of care they provide.

Based on prior research findings, Kanter’s theory was used to investigate the influence of practice environment and leadership style on NP interprofessional collaboration and the quality of care in acute care settings. The allocation of structural power and resources within an organization has been shown to significantly influence individual performance (Kanter, 1968). Individuals who have easy access to essential resources and possess the authority to engage in decision-making tend to exhibit enhanced initiative and a stronger sense of responsibility, leading to more effective interprofessional collaboration with individuals and teams and better quality of care. In this study, the authors conceptualized the practice environment and leadership styles as elements of structural power and resources within health care organizations, while interprofessional collaboration and quality of care were regarded as indicators of individual performance. These factors were hypothesized to influence both collaborative practices and quality of care significantly.

## Methods

### Study Design, Setting, and Participants

In this study, a cross-sectional design and nation-wide survey were used. Data were collected using an online survey from August 2, 2023, to January 31, 2024. The participants were recruited from the Taiwan Association of Nurse Practitioners by systematic sampling. The inclusion criteria were: being a certified national nurse practitioner and having a minimum of one year of experience working in an acute care setting. First, TANP assisted with disseminating emails containing information on the study purpose, procedures, and participant rights and a link to the online survey to all eligible NPs in their database. Those willing to participate completed the questionnaires on the online survey platform. The multiple linear regression analysis employed in this study required a minimum sample size of 199 participants, as calculated using G*Power 3.1.9.2 software with parameters set at an effect size (*f²*) of 0.10, a significance level (α) of .05, a power of 0.80, and a total of eight predictors. After accounting for an anticipated response rate of approximately 20%, the minimum sample size target was raised to 239. Ultimately, a total of 1,217 eligible NPs participated in the online survey, with 19 subsequently excluded due to incomplete demographic data. Thus, the final analysis was conducted using valid data collected from 1,198 participants.

### Measurement

#### Participant Demographic Characteristics

The demographic characteristics data for the participants were collected using a self-administered questionnaire that included questions covering age, gender, marital status, educational background, employment status, hospital level of practice, daily working hours, years of experience as an NP, level on the professional career ladder, number of patients cared for daily, and annual salary.

#### Interprofessional Collaboration and Quality of Care

Interprofessional collaboration and quality of care were measured using the Provider-Perceptions of Team Effectiveness questionnaire (Provider-PTE) originally developed by Kilpatrick et al. (2019). This questionnaire consists of 43 self-administered items scored using a 6-point Likert scale ranging from 1 (*very dissatisfied*) to 6 (*very satisfied*). The two major sections of the questionnaire are PTE-Overall and Outcomes. The PTE-Overall includes role clarity, boundary work, team meetings, and processes. In this study, the revised PTE-Overall section (19 items) was used to assess interprofessional collaboration, while the revised Outcomes section (7 items) was used to assess quality of care, with higher scores indicating, respectively, higher levels of team functioning and quality of care in practice. The Provider-PTE has previously demonstrated good reliability and validity (Kilpatrick et al., 2019; Kilpatrick et al., 2021). After receiving permission from the developer, a back translation procedure was used to translate the Provider-PTE into Chinese for use in this study. The content validity of the translated version was assessed by two senior nursing experts, with the results indicating favorable content validity. In this study, the Cronbach’s alpha coefficient for the Chinese version of PTE-Overall was .93, while the reliability for the quality of care section was .87.

#### Practice Environment

Practice environment was assessed using the Nurse Practitioner Acute Care Organizational Climate Questionnaire (NP-ACOCQ), which was adapted by Gigli et al. (2024) from the NP-ACOCQ developed by Poghosyan et al. (2019). The NP-ACOCQ comprises 29 items, each of which is scored on a 4-point Likert-type scale ranging from 1 (*strongly disagree*) to 4 (*strongly agree*). The questionnaire encompasses four subscales designed to evaluate the various domains of organizational climate, including NP-physician relations, professional visibility, NP-administration relations, and independent practice and support, with higher scores indicating a more favorable perception of the NP organizational climate. The NP-ACOCQ has demonstrated satisfactory reliability and validity in research conducted in Taiwan and other countries (Gigli et al., 2024; Ho et al., 2021). In this study, the Cronbach’s alpha coefficients for each subscale were .94 for NP-physician relations, .87 for professional visibility, .83 for NP-administration relations, and .82 for independent practice and support.

#### Manager Leadership Style

The Multifactor Leadership Questionnaire–Form 6S (MLQ–6S) was used to assess leadership style. The MLQ-6S comprises 21 items, each of which is rated on a 5-point Likert scale ranging from 1 (*strongly disagree*) to 5 (*strongly agree*). The MLQ-6S encompasses seven distinct leadership styles, including idealized influence, inspirational motivation, intellectual stimulation, individual consideration, contingent reward, management-by-expectation, and laissez-faire (Bass & Avolio, 1992), with higher scores indicating a greater perception of the related leadership style. Both the MLQ-6S and the Chinese version of MLQ6-S have reported good reliability and validity in previous studies (Hsu et al., 2022; Middleton et al., 2023; Moon et al., 2019). In this study, Cronbach’s alpha values for the various leadership styles were .93 for idealized influence, .90 for inspirational motivation, .95 for intellectual stimulation, .87 for individual consideration, .66 for management-by-expectation, .74 for laissez-faire, and .92 for contingent reward.

### Ethical Considerations

All of the procedures used in this study were approved by the institutional review board of the Ditmanson Medical Foundation, Chia-Yi Christian Hospital (Approval number: IRB2022116).

### Statistical Analysis

Descriptive statistics, including percentages, means, and *SD*, were used to summarize the distributions of the study variables. Independent *t*-test, analysis of variance, and the Pearson product-moment correlation coefficient (*γ*) were used to examine the correlations between independent variables and, respectively, interprofessional collaboration and quality of care. Multiple regression analysis was performed to identify potential factors of influence on outcomes, with significant variables incorporated into the multiple regression model using the stepwise selection method. The multicollinearity of the regression model was examined using a variance inflation factor (VIF) threshold of <10 (Thompson et al., 2017), with the significance level set at .05, with two-tailed tests. All of the statistical analyses were conducted using IBM SPSS Version 24 (IBM Corp., Armonk, NY, USA).

## Results

### Demographic Characteristics, Interprofessional Collaboration, and Quality of Care

The distributions of demographic variables, interprofessional collaboration, and quality of care are presented in Table 1. Most of the participants were female (93.50%), married (66.90%), and held either a college or university degree (79.40%). The average practice experience was 8.32 years (*SD*=4.59), and the average daily work duration was approximately 9.08 hours (*SD*=1.20). The participants managed care for 13.63 patients per day (*SD*=8.65), with a mean patient age of 42.8 years (*SD*=6.58). Most engaged in shift work (54.3%) and practiced in either a regional hospital (47.10%) or a medical center (33.90%). The average score on the professional career ladder (NP1-NP5 scale: 1–5) was 2.27 (*SD*=1.14), and the average annual salary was 81.00 (10,000 NTD; *SD*=14.67).

**Table 1:** Participant Demographic Characteristics (N = 1,198)

Variable	*n* (%)
Age (years; *M* and *SD*)	42.8 (6.58)
Gender	
Female	1,120 (93.5)
Male	78 (6.5)
Marital status	
Married	801 (66.9)
Unmarried	397 (33.1)
Educational level	
College/university	951 (79.4)
Graduate school or above	247 (20.6)
Working status	
Fixed shift	548 (45.7)
Rotated shifts	650 (54.3)
Hospital level	
Medical center	406 (33.9)
Regional hospital	564 (47.1)
District hospital	228 (19.0)

	*M* (*SD*)
Daily working hours	9.08 (1.20)
Years of NP experience	8.32 (4.59)
Career ladder level (NP1-NP5)	2.27 (1.14)
Number of daily patients cared for	13.63 (8.65)
Annual salary (10,000 NTD)	81.00 (14.67)
PTE	
Quality of care	31.97 (5.00)
Interprofessional collaboration	82.98 (12.66)
Practice environment	
Professional visibility	11.29 (2.22)
Administration relation	22.47 (5.66)
Physician relation	20.74 (2.94)
Independent practice social support	25.60 (3.63)
Leadership styles	
Idealized influence	6.05 (2.89)
Inspiration motivation	5.57 (2.89)
Intellectual stimulation	5.30 (3.03)
Individual consideration	5.61 (2.84)
Management by expectation	7.29 (2.32)
Laissez faire	6.11 (2.41)
Contingent reward	5.29 (3.05)

*Note.* NTD = New Taiwan Dollar; PTE = Perceptions of Team Effectiveness Questionnaire.

The mean scores for quality of care and interprofessional collaboration were 31.97 (*SD*=5.00) and 82.98 (*SD*=12.66), respectively. Of the organizational climate subscales, independent practice social support earned the highest mean score of 25.60 (*SD*=3.63). Of the leadership style subscales, management-by-expectation leadership style earned the highest mean score.

### Correlations Among Demographic Variables, Interprofessional Collaboration, and Quality of Care

Significant associations were found among interprofessional collaboration, quality of care, and demographic variables such as age, marital status, working shift, hospital level, NP experience, and number of daily patients (Table 2). The significantly positive correlations found among organizational climate, leadership style subscales, interprofessional collaboration, and quality of care are shown in Table 3 (*p*<.01).

**Table 2:** Correlations Among Demographic Characteristics, Interprofessional Collaboration, and Quality of Care (N = 1,198)

Variable	Interprofessional Collaboration			Quality of Care		
	Mean (*SD*)	*t*/*F*/*r*	*p*/Post Hoc	Mean (*SD*)	*t*/*F*/*r*	*p*/Post Hoc
Age (year)	42.80 (6.59)	.14***	<.001	42.80 (6.59)	.14***	<.001
Gender						
Female	83.01 (12.56)	0.39	.70	32.01 (4.95)	0.96	.34
Male	82.44 (14.02)			31.45 (5.56)		
Marital status						
Married	83.77 (12.54)	3.09**	.002	32.20 (4.94)	2.23*	.03
Unmarried	81.38 (12.76)			31.52 (5.07)		
Educational level						
College/university	83.03 (12.56)	−0.27	.79	31.96 (4.91)	0.16	.87
Graduate school or above	82.78 (13.06)			32.02 (5.33)		
Working status						
Fixed shift	84.42 (12.45)	−3.63***	<.001	32.45 (5.18)	−3.01**	.003
Rotated shifts	81.76 (12.71)			31.58 (4.81)		
Hospital level^a, b^						
① Medical center	85.27 (12.04)	14.77***	<.001	32.90 (4.80)	13.38*	<.001
② Regional hospital	82.65 (12.62)		①>③, ②>③, ①>②	31.76 (5.12)		①>③, ②>③
③ District hospital	79.71 (13.05)			30.86 (4.75)		
Daily working hours	9.08 (1.20)	−.06	.05	9.08 (1.20)	−.04	.15
Years of NP experience	8.32 (4.59)	.07*	.01	8.32 (4.59)	.08**	.005
Career ladder level (NP1-NP5)	2.27 (1.14)	.07*	.02	2.27 (1.14)	.01	.86
Number of daily patients cared for	13.63 (8.65)	.12**	<.01	13.63 (8.65)	−.07*	.01
Annual salary (10,000 NTD)	81.03 (14.67)	.15***	<.001	81.03 (14.67)	.13***	<.001

*Note.* NTD = New Taiwan Dollar.

*p<.05. **p<.01. ***p<.001.

**Table 3:** Correlations Among Practice Environment, Leadership Style, Quality of Care, and Interprofessional Collaboration (N = 1,198)

Variable	Interprofessional Collaboration	Quality of Care
Practice environment		
Professional visibility	.57**	.47**
Administration relation	.54**	.43**
Physician relation	.59**	.49**
Independent practice social support	.53**	.43**
Leadership style		
Idealized influence	.43**	.35**
Inspiration motivation	.40**	.33**
Intellectual stimulation	.39**	.32**
Individual consideration	.38**	.31**
Management by expectation	.37**	.31**
Laissez faire	.32**	.21**
Contingent reward	.36**	.30**

**p<.01.

### Factors Influencing Interprofessional Collaboration

Interprofessional collaboration was shown to be influenced positively by organizational climate and leadership style. Specifically, factors such as physician relations, professional visibility, management-by-expectation, social support for independent practice, and the idealized influence leadership style were associated positively with enhanced interprofessional collaboration (*p*<.05). Furthermore, older age improved interprofessional collaboration (*p*<.01), whereas higher daily patient load decreased interprofessional collaboration (*p*<.05). The results of the multiple regression analysis indicate 47.6% of the variance in interprofessional collaboration was explained by these factors (Table 4). The VIF for the factors ranged from 1.02 to 2.09, suggesting no collinearity issues were exhibited in the regression model.

**Table 4:** Factors Associated With Interprofessional Collaboration and Quality of Care (N = 1,198)

Variable	B	*SE*	β	*t*	*p*	Δ*R* ^2^
**Interprofessional collaboration**						
Constant	18.00	2.78	—	6.48***	<.001	—
Practice environment						
Physician relation	1.25	0.13	.29	9.59***	<.001	.350
Professional visibility	1.46	0.16	.26	9.06***	<.001	.083
Leadership style						
Management by expectation	0.48	0.15	.09	3.24**	.001	.015
Hospital level (ref: district hospital)						
Medical center	2.07	0.57	.08	3.63***	<.001	.009
Practice environment						
Independent practice social support	0.46	0.10	.13	4.45***	<.001	.008
Age	0.14	0.04	.07	3.34**	.001	.005
No. daily patients cared for	−0.08	0.03	−.05	−2.55*	.010	.003
Leadership style						
Idealized influence	0.31	0.13	.07	2.47*	.010	.003
Adjusted *R* ^2^	.47 (*F*=134.49, *p*<.001)					
**Quality of care**						
Constant	8.89	1.19	—	7.50***	<.001	—
Practice environment						
Physician relation	0.43	0.06	.25	7.41***	<.001	.240
Professional visibility	0.48	0.07	.21	6.87***	<.001	.054
Leadership style						
Management by expectation	0.31	0.07	.14	4.44***	<.001	.012
Hospital level (ref: district hospital)						
Medical center	1.02	0.25	.10	4.07***	<.001	.011
Age	0.07	0.02	.10	4.04***	<.001	.007
Practice environment						
Independent practice social support	0.16	0.05	.11	3.36**	.001	.005
Leadership style						
Laissez faire	−0.25	0.07	−.12	−3.85***	<.001	.006
Contingent reward	0.13	0.05	.08	2.65**	.008	.004
Adjusted *R* ^2^	.34 (*F*=76.47, *p*<.001)					

*Note.* SE = standard error.

*p<.05. **p<.01. ***p<.001.

### Factors Influencing Quality of Care

Significant influences on quality of care were identified in relation to organizational climate and leadership style (Table 4). Specifically, physician relations, professional visibility, management-by-expectation, and social support for independent practice were found to influence quality of care positively (*p*<.01). Moreover, quality of care was shown to be enhanced by working in a medical center (*p*<.01) and being of older age (*p*<.01). The multiple regression model accounted for 33.9% of the variance in quality of care (*p*<.01). Moreover, the VIF of less than 5 (1.01–2.09) for all factors indicates the regression model as free from collinearity issues.

## Discussion

The findings of this study indicate that practice environment, including physician relations, professional visibility, independent practice social support, and management-by-expectation leadership style, are significant variables that positively affect interprofessional collaboration and quality of care. Together, these variables explained 45.6% of the variance in interprofessional collaboration and 31.1% of the variance in quality of care. These findings support Kanter’s theory, which posits that practice environment and leadership style facilitate the access of NPs to organizational resources and authority, subsequently allowing NPs to achieve improved interprofessional collaboration and higher-quality outcomes. Hence, improving the supportive practice environment and adopting an appropriate leadership style are essential to enhancing NP interprofessional collaboration and their quality of care.

The findings show the participants perceived that having a favorable practice environment could enhance interprofessional collaboration, which is consistent with previous studies (Donelan et al., 2020; Ho et al., 2021; Pérez-Vallejo & Fernández-Muñoz, 2020). Notably, improved relationships with physicians within the practice environment were the main contributor to improved collaboration. This may reflect strengthened physician relations as likely to enhance NP perceptions of organizational support, thereby motivating NPs to assert their clinical expertise and engage with health care professionals. For example, Ho et al. (2021) demonstrated that robust organizational support enhances positive interactions and cooperation between NPs and physicians. Similarly, Poghosyan et al. (2022) suggested that healthy interactions among health care team members are indicative of mutually beneficial relationships between NPs and physicians. Generally, in Taiwan, as every physician teams up with NPs to care for patients, the NP-physician relationship influences the level of interprofessional collaboration, with positive physician relations potentially encouraging NPs to communicate and coordinate effectively with physicians and leading, ultimately, to well-integrated professional collaboration.

Moreover, the findings indicate the relationship between physicians and NPs may also increase the quality of care provided, which echoes the findings of previous studies (Barnett et al., 2022; Schirle et al., 2020). As mentioned, NPs primarily provide hospital-based care and practice collaboratively with physicians to manage patient treatment. A satisfactory physician–NP relationship that facilitates collaboration and the sharing of practice responsibilities should ensure patients receive appropriate and timely treatment and, ultimately, enhance quality of care (Poghosyan et al., 2022). The evidence of prior studies suggests NPs who report stronger relationships with physicians are more likely to provide higher-quality care (Poghosyan, Ghaffari, Liu, & Friedberg, 2020) and practice effectively (Schirle et al., 2020). Moreover, (Poghosyan, Ghaffari, Liu, & McHugh (2020) noted that favorable practice conditions significantly promote NP quality of care. Consequently, in this study, the participants identified supportive physician relationships within organizations as a means of improving care quality.

In addition, the findings of this study indicate professional visibility within the practice environment to be positively correlated with interprofessional collaboration, which is consistent with previous studies (Donelan et al., 2020; Etherington et al., 2021). Although guidelines for NP practice have been established, hospitals generally regulate the actual practice of their NPs based on the demands of health care organizations (Luo et al., 2021). In Taiwan, clinical decision-making often prioritizes the opinions of physicians over collaborative consultations with NPs. This may obscure the professional roles of NPs within interprofessional teams, leading to role overlap and a diminishing of their unique contributions (Hsu et al., 2022). Consequently, NPs may be less inclined to view physicians as collaborators within interprofessional teams, which may then decrease the effectiveness of interprofessional collaboration. Therefore, establishing clearly defined role delineations for NPs has the potential to alleviate role ambiguity and reduce the incidence of overlapping responsibilities, thus strengthening the effectiveness of interprofessional collaboration.

Professional visibility within the practice environment was also shown to enhance quality of care in this study, which is in line with the previous studies (Abraham et al., 2021; Kilpatrick et al., 2021). This correlation may be attributed to the negative impact of ambiguous professional roles on NP functioning, which may impact the process of care. In Taiwan, the scope of NP practice is often shaped by hospital-level guidelines that often do not clearly identify the professional role of NPs in patient care (Hsu et al., 2022). Enhancing professional role clarity within health care teams may facilitate more effective coordination between NPs and physicians. Schirle et al. (2020) also pointed out that a lack of understanding regarding professional roles can create barriers to optimal practice. Also, Kilpatrick et al. (2021) emphasized that enhanced role clarity was associated with improved care outcomes. The overlap in scopes of practice between NPs and physicians highlights the necessity for clear professional roles in health care delivery (Donelan et al., 2020; Ho et al., 2021). Consequently, NPs may experience insufficient professional visibility within the health care team, generating adverse impacts on the quality of care.

The findings of this study also indicate that social support for independent practice within the practice environment positively influences interprofessional collaboration. This may contribute to the inadequate organizational support experienced by NPs, particularly in the realm of independent practice. The restrictive practice model implemented by hospitals may limit the ability of NPs to practice independently within their regulated scope of practice, thereby decreasing interprofessional collaboration efficacy (Ho et al., 2021). Organizational support is pivotal in promoting effective teamwork and decision-making within interprofessional teams. Moilanen et al. (2020) emphasized interprofessional collaboration as a critical objective for health care organizations that necessitates enhanced resources and actions in practice. Furthermore, providing adequate support has the potential to facilitate efficient team-based practice (Huang et al., 2024). Consequently, NPs who perceive sufficient social support for independent practice from health care organizations tend to have enhanced role functionality and better interprofessional collaboration performance.

Social support for independent practice within the practice environment was also found to positively influence quality of care, which aligns with the findings of previous studies (Ho et al., 2021; Poghosyan, Ghaffari, Liu, & McHugh, 2020). This may be due to social support for independent practice facilitating greater NP access to organizational support and resources, and thus promoting their practice capabilities. NPs who perceive robust organizational support are more likely to demonstrate higher levels of care quality in patient care (Poghosyan, Ghaffari, Liu, & McHugh, 2020). Schirle et al. (2020) noted that adequate independent practice may allow NPs to fully utilize their functions and deliver improved patient care. Furthermore, NPs with stronger perceptions of organizational support demonstrate enhanced performance in responding to feedback (Ho et al., 2021). In other words, NP practice effectiveness depends largely on organizational support, which facilitates the delivery of higher-quality care.

Moreover, leadership style was found in this study to positively influence interprofessional collaboration and quality of care, which is consistent with the findings of previous research (Alanazi et al., 2023; Hsu et al., 2022; Moilanen et al., 2020). Also, the management-by-expectation style was found to enhance interprofessional collaboration, which may be due to this management style providing to NPs greater autonomy and empowerment through the authorization to them of routine decision-making. Thus, managers who adopt a management-by-expectation leadership style may place greater emphasis on the demands of their NPs to ensure effective team-based practices. Specifically, the professional roles of NPs are likely to be enhanced through the perception of autonomy and empowerment, which in turn improves the practice of NPs. As revealed in previous studies, adequate autonomy and empowerment are important to enhancing the practice of NPs, which then can foster greater confidence in their rights and entitlements (Hsu et al., 2022; Luo et al., 2021). The management-by-expectation leadership approach may also encourage NPs to more actively share their recommendations and communicate their professional perspectives with team members, especially physicians, facilitating more efficient interprofessional collaboration and higher overall performance.

The findings further show that the idealized influence leadership style positively affected interprofessional collaboration, which aligns with the results of prior research (Folkman et al., 2019; Hsu et al., 2022; Lin et al., 2024). This may account in part for the positive effects of idealized influence on the practice of NPs. NPs are more inclined to trust and respect managers who adopt an idealized influence leadership style, motivating them to align more closely with management objectives and adopt NP practices aimed at achieving care practice. The existence of this mechanism has support in the literature. For example, enhancements in clinical performance may arise from the motivation individuals derive from the trust and integrity demonstrated by their managers (Allan & Rayan, 2023). Folkman et al. (2019) emphasized the necessity for managers to provide support and facilitate change within the work environment to enhance interprofessional collaboration. Similarly, Hsu et al. (2022) demonstrated that the idealized influence leadership style can enhance interprofessional collaboration. Moreover, managers who adopt an idealized influence style are more likely to show stronger beliefs about the professional role of NPs, thereby facilitating the effectiveness of interprofessional collaboration. This support may engender trust in and value for NPs from their managers, resulting in stronger connections between NPs and team members, and ultimately improving the performance of interprofessional collaboration in practice.

Several environmental variables were also identified in this study related to NPs that were associated with interprofessional collaboration and quality of care (Carthon et al., 2022; Poghosyan, Ghaffari, Liu, & McHugh, 2020). This may be attributable to older individuals frequently possessing more practical experience than younger ones, which enables them to effectively address challenges in clinical care and support each other, leading to enhanced interprofessional collaboration and quality of care. Furthermore, the participants practicing in medical centers reported superior levels of interprofessional collaboration and care quality. This observation is attributed to differences in the level of organizational support received at medical centers and hospitals, respectively. In addition, increased patient load was found to negatively impact interprofessional collaboration. This may be due to higher patient care demands reducing practice effectiveness and interprofessional collaboration (Abraham et al., 2021).

### Limitations

This study was affected by several limitations. First, the cross-sectional design employed limits the ability to draw conclusions regarding causality. Second, most of the participants were recruited from acute care settings, which may limit the generalizability of the findings. Third, the data used in this study were collected via an online survey, which might have introduced response bias.

### Conclusions

The findings of this study may reduce the current knowledge gaps related to NP interprofessional collaboration and the quality of care they provide in Taiwan. The primary factors influencing these two outcomes were found to be the practice environment, which encompasses relationships with physicians and professional visibility, and the management-by-expectation leadership style.

### Implications for Practice

Based on the findings of this study, health care organizations should focus on optimizing the practice environment by fostering positive relationships among physicians and ensuring clear professional visibility. Health care organizations should modify guidelines and protocols throughout the Hospital Level Nurse Practitioner Practice Committee to empower NPs to make patient care decisions, engage in supportive care management tasks (e.g., patient follow-up, tube management, and wound treatment), and communicate clearly the roles of NPs to team members, particularly in relation to physicians. For example, physicians should hold regular meetings and engage in open discussions with NPs to clearly identify their demands and duties in clinical practice and to promote their involvement in decision-making processes. These initiatives may also improve the sense of organizational support in independent practice among NPs, thus enhancing their interprofessional collaboration and the overall quality of care they provide. Furthermore, health care organizations should promote the adoption and use of the management-by-expectation leadership style to optimize the practice of NPs. Within the expectation management leadership style framework, characterized by a focus on adherence to clinical guidelines and established work standards, NPs may find it more easy to practice with precisely defined protocols. This approach may be expected to facilitate the authority of NPs, enabling them to actively participate in interprofessional decision-making processes together with physicians in clinical settings and, ultimately, enhancing their interprofessional collaboration and elevating the quality of patient care they provide.
